# Supplementary material for: TH17 cells promote CNS inflammation by sensing danger signals via Mincle
Source: Nat Commun. 2022 May 3;13:2406. doi: 10.1038/s41467-022-30174-1 (PMC9064974; doi:10.1038/s41467-022-30174-1)
Supplement: Supplementary file 1 — Supplementary Information [file 41467_2022_30174_MOESM1_ESM.pdf]

Supplementary Information

TH17 cells promote CNS inflammation by sensing danger signals via Mincle

Quanri Zhang et al.
Corresponding authors: Xiaoxia Li, Laura E. Nagy

Supplementary Fig1. mRNA expression of CLRs in EAE CD4+ and Th cells

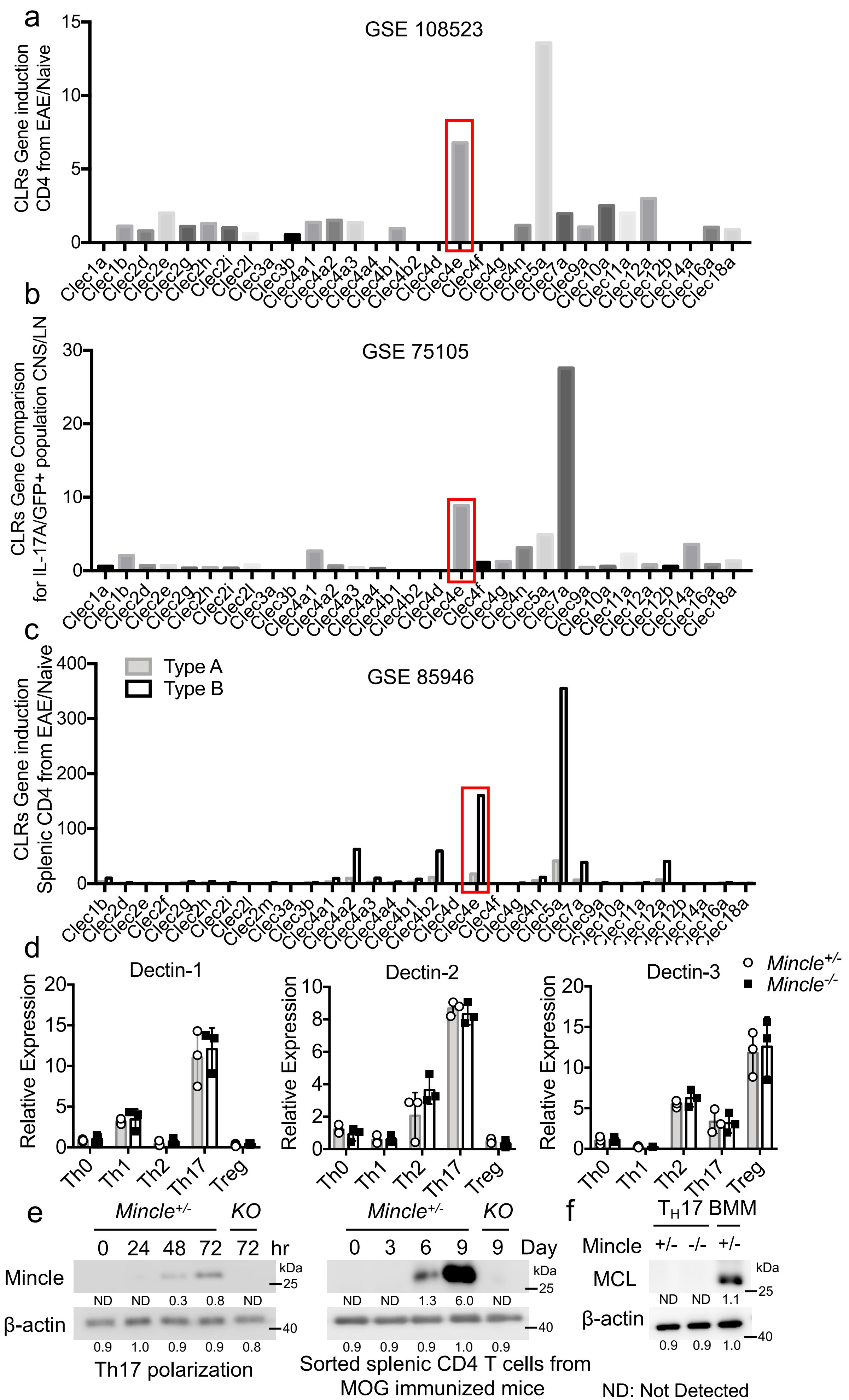

Supplementary Fig 1. mRNA expression of CLRs in EAE CD4+ and T_H cells **(a-c)** CLRs mRNA induction in CD4 T or IL-17+ cells were compared in EAE and naïve mice from the public microarray database. **(d)** Expression of Clec7a(Dectin-1), Clec4n(Dectin-2) and Clec4d(Dectin-3) on T_H0, T_H1, T_H2, T_H17 and Treg cells after 3 days polarization was measured by Real-time PCR, n=3 biological replicates. Relative mRNA expression was compared by setting the lowest expression amount to an arbitrary value of 1. **(e)** Western blot analysis of Mincle expression from Naïve CD4 T cells cultured under T_H17 polarization conditions (left) and sorted splenic CD4 T cells from MOG (right) at indicated times. **(f)** Western blot analysis of MCL expression in polarized *Mincle*$^{+/-}$ and *Mincle*$^{-/-}$ T_H17 cells, bone marrow macrophages were included as a positive control. Data is representative of 3 independent experiments (**e**,**f**). Density values measured using Image J for the representative blot shown, ND not detected. Data are represented as mean $\pm$ SD.

Supplementary Fig2. Reduced active EAE in CD4-cre Mincle$^{f/f}$ mice

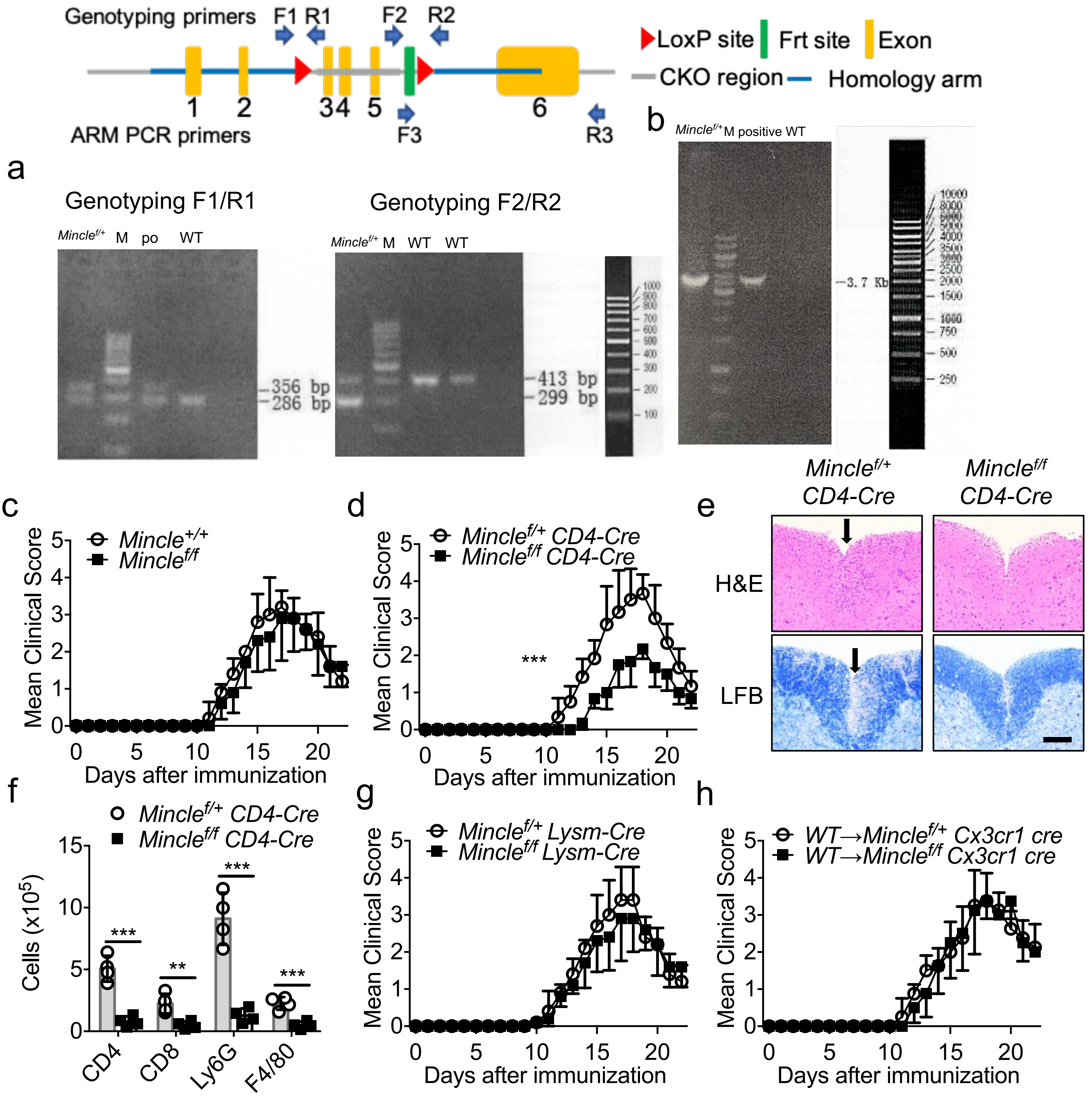

Supplementary Fig 2. Reduced EAE in CD4-cre *Mincle*$^{f/f}$ mice but not in L*ysM-Cre Mincle*$^{f/f}$ or MOG reactive T_H17 adoptive transferred *Cx3Cr1-cre Mincle*$^{f/f}$ mice **(a)** Genotyping of *Mincle*$^{f/+}$ mice by PCR followed by agarose gel electrophoresis. Data is representative for at least 3 independent experiments. **(b)** ARMS PCR (Amplification Refractory Mutation System PCR) to screening for the expected 3.7 Kb fragment insertion. Data is representative for 2 independent experiments. **(c)** EAE clinical scores of *Mincle*$^{f/f}$ and *Mincle*$^{+/+}$ mice induced active EAE, n=5 mice. **(d)** EAE clinical scores of *Mincle*$^{f/+}$*CD4-Cre* and *Mincle*$^{f/f}$*CD4-Cre* mice induced active EAE, n=5 mice. **(e)** Hematoxylin and eosin (H&E) staining (upper panels) and Luxol fast blue staining (lower panels) of lumbar spinal cords from *Mincle*$^{f/+}$*CD4-Cre* and *Mincle*$^{f/f}$*CD4-Cre* EAE mice harvested at the peak of disease. Scale bar, 100µm. Arrows in the upper panel indicate inflammatory cells infiltration, and arrows in the lower panel indicate demyelination area. Representative data are shown for n = 4. **(f)** Absolute numbers of CNS-infiltrating cells were measured in *Mincle*$^{f/+}$*CD4-Cre* and *Mincle*$^{f/f}$*CD4-Cre* EAE mice at the peak of disease by flow cytometry with indicated antibodies, n=4 biological replicates. **(g)** EAE clinical scores of L*ysM-Cre Mincle*$^{f/f}$ mice induced active EAE, n=5 mice. **(h)** Mean clinical score of EAE mice induced by adoptive transfer of WT MOG-reactive T_H17 cells to *Cx3Cr1-cre Mincle*$^{f/f}$ mice, n = 5 mice. *P < 0.05, ***P<0.001 (Two sided student's t test **f**). **P < 0.01 (Two-way ANOVA, **c, d ,g and h**). Data are represented as mean ± SD. Exact *P* values for asterisks (from left to right): d <0.0001 f 0.00026 0.0064 0.00037 0.00099

Supplementary Fig 3. Modulation of T cell survival occurs in the CNS, but not in the periphery

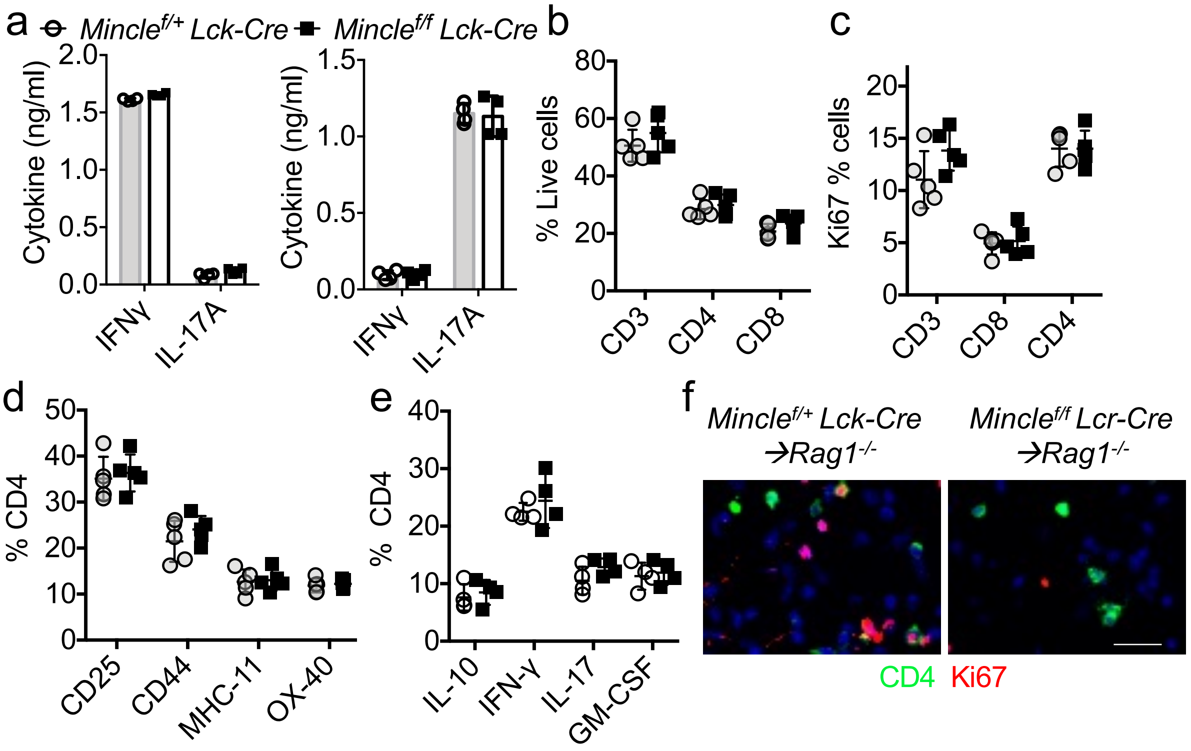

Supplementary Fig 3. Modulation of T cell survival occurs in the CNS, but not in the periphery **(a)** ELISA assay for the IL-17A and IFN-γ in the supernatant of cells from the draining lymph nodes of mice 10 d after immunization with MOG_{35-55} followed by *ex vivo* restimulation in the presence of MOG_{35-55} and either IL-12 (**left**) or IL-23 (**right**), n=4 biological replicates. **(b)** Percentage of total CD3, CD4 and CD8 cells in lymph nodes from mice 10 day after immunization with MOG measured by flow cytometry, n=5 biological replicate. **(c-e)** Percentages of $Ki67^+$(C), subsets with surface activation markers **(d)** and cytokine producing cells **(e)** in CD4 T cells were measured from mice 10 d after immunization with MOG, n=5 biological replicates. **(f)** Ki-67/CD4 staining of spinal cords from adoptive transfer experiments from day 9 after transfer and percentage of CD4+ T cells co-staining with Ki-67. Scale bar, 10μm. Red, Ki67+. Green, CD4+. Representative data are shown for n = 4. (Two sided student's t test for **a-e**) Data are represented as mean ± SD.

Supplementary Fig 4. TDB promotes Th17 polarization through Mincle signal

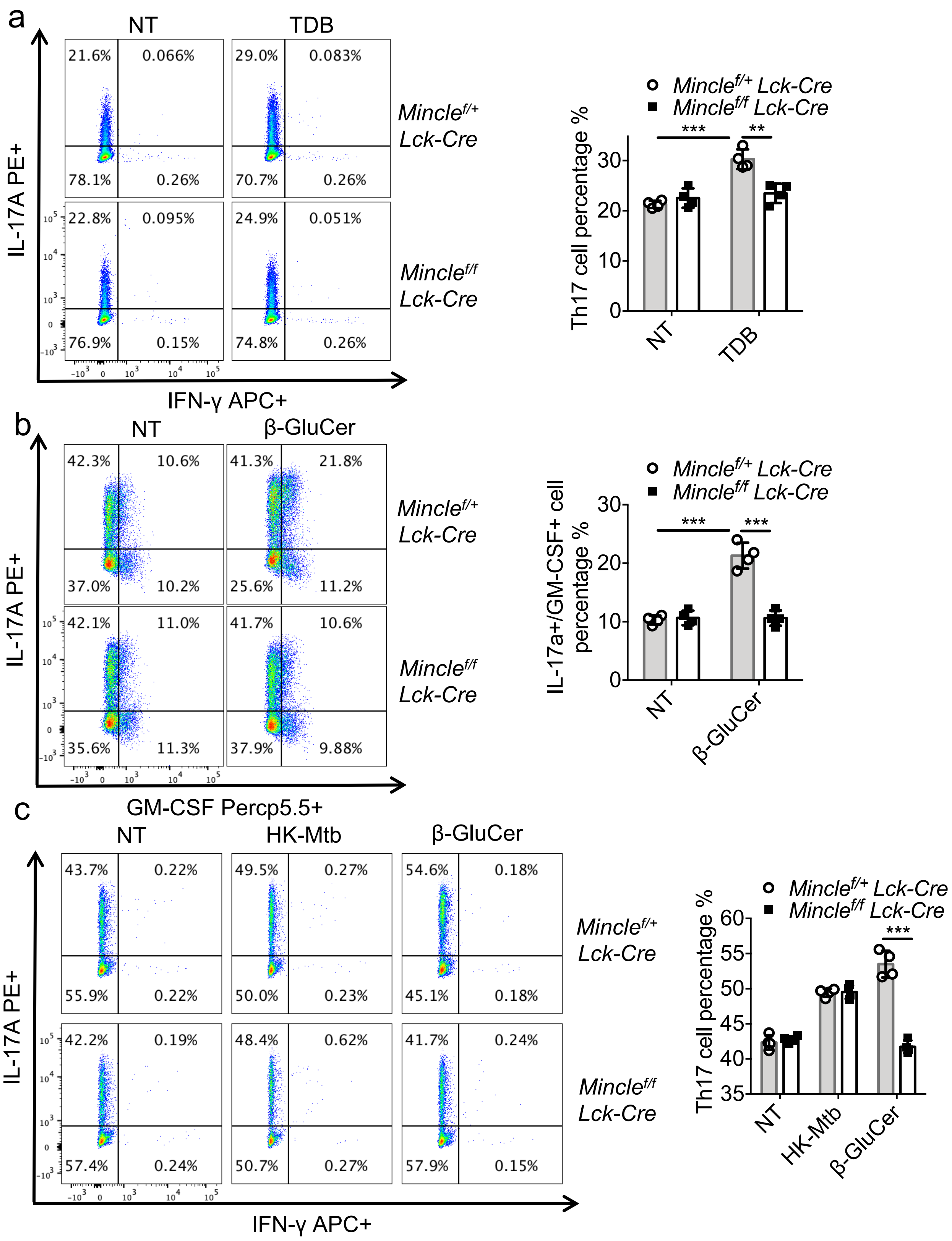

Supplementary Fig 4. *Mincle* ligands promotes inflammatory T_H17 differentiation **(a)** Intracellular cytokine staining of IL17A and IFNγ following 3 days T_H17 differentiation with or without 20ug/ml TDB and analyzed by flow cytometry, n=4 biological replicates. **(b)** Polarized T_H17 were rest for 2 days with IL-2(2ng/ml) and re-stimulated with CD3/CD28 with or without β-GluCer (5μg/ml) for another 3 days, followed by intracellular cytokine staining for IL17A and GMCSF and analyzed by flow cytometry, n=4 biological replicates. **(c)** Flow cytometric analysis of IL-17A and IFNγ following 3 days of T_H17 polarization with vehicle (PBS), HK-Mtb (10μg/ml) or β-GluCer (5μg/ml), n=4 biological replicates. **p<0.01, ***p<0.001 (Two sided student's t test). Data are represented as mean ± SD. Exact *P* values for asterisks (from left to right): **a** 0.00013 0.0026 **b** 0.00009 0.00017 **c** 0.00003

Supplementary Fig 5

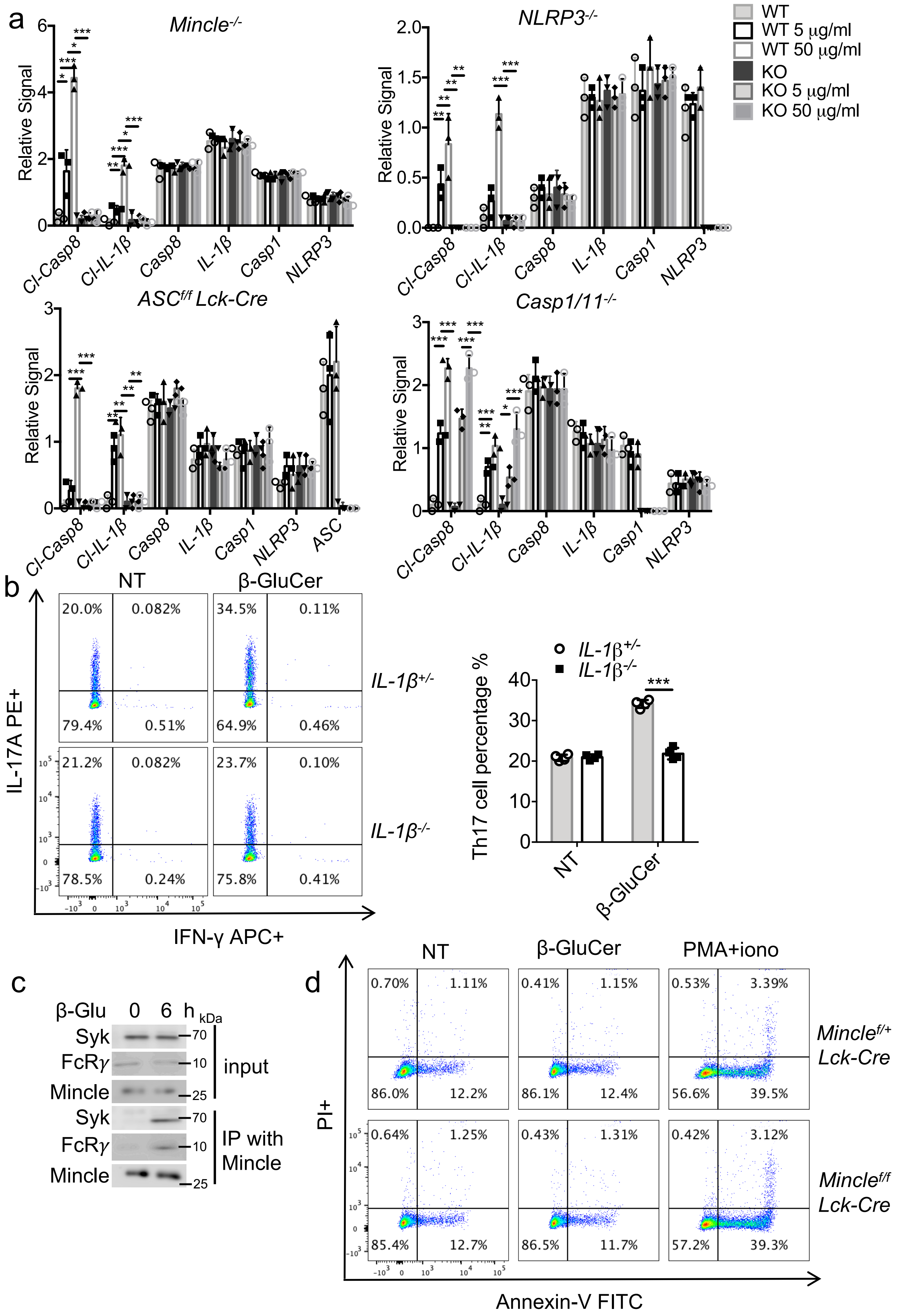

Supplementary Fig 5. β-glucosylceramide could activate a *Mincle*-FcRγ-Syk-dependent IL-1β production and enhance the T_H17 proliferation. **(a)** Quantification of western blot band **(Fig 5a-d)** with Image J and the signal were normalized to β-actin, n=3 biological replicates. **(b)** Flow cytometry analysis of polarized *IL-1b*$^{+/-}$ and *Il-1b*$^{-/-}$ T_H17 polarized with or without β-glucosylceramide (5μg/ml), n=4 biological replicates. **(c)** Cell lysates from T_H17 treated with β-glucosylceramide (50μg/ml) were subjected to immunoprecipitation with anti-*Mincle*, followed by western analysis with the indicated antibodies. Data is representative for at least 3 independent experiments. **(d)** Annexin V/Propidium Iodide cell viability assay were performed via flow cytometry on T_H17 cells polarized with β-glucosylceramide (5μg/ml). Cells treated with PMA and ionomycin for 4 hours were used as a positive control. Data were representative of 3 different individual experiments. *P < 0.05, **P<0.01, ***P<0.001 (Two sided student's t test, **a, b**). Data are represented as mean ± SD. Exact *P* values for asterisks (from left to right): **a** 0.0213 0.00005 0.0214 0.00005 0.0080 0.0002 0.0341 0.0002, 0.0080 0.0091 0.0080 0.0091 0.0006 0.0003, 0.00001 0.00001 0.0034 0.0036 0.0034 0.0036, 0.0004 0.00003 0.00012 0.00006 0.0018 0.0009 0.0147 0.0018 **b** <0.0001

Supplementary Fig 6. β-Glucosylceramide or AMP-DNM administration did not affect EAE in *Mincle$^{f/f}$ CD4-Cre* Mice

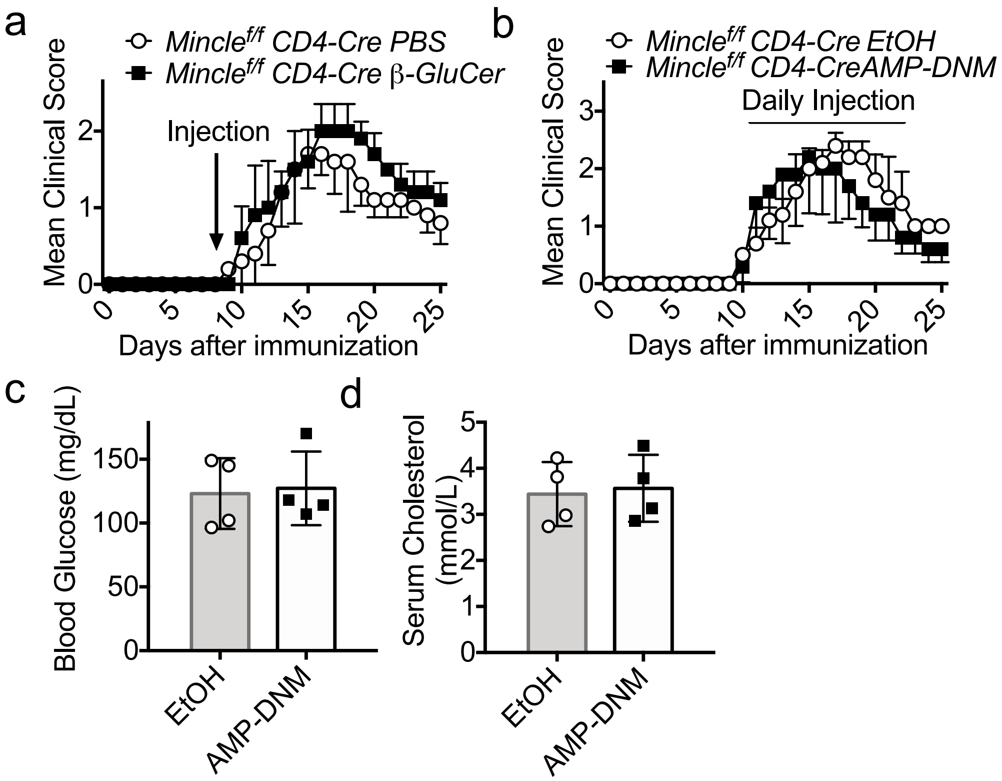

Supplementary Fig 6. β-Glucosylceramide or AMP-DNM administration did not affect EAE in *Mincle$^{f/f}$CD4-Cre* Mice **(a)** EAE clinical scores of *Mincle$^{f/f}$CD4-Cre* mice treated with β-glucosylceramide (150μg/mice) or vehicle during EAE induction, n=5 mice. **(b)** EAE clinical scores of *Mincle$^{f/f}$CD4-Cre* mice intraperitoneally injected daily with AMP-DNM (1mg/kg) or ethanol dissolved in PBS at the onset of the EAE, n=5 mice. **(c-d)** Blood glucose and serum cholesterol concentration were measured from the EAE mice (**Fig. 5f**) that treated with AMP-DNM or vehicle, n=4 biological replicates. (Two sided student's t test, **c, d**). (Two-way ANOVA, **a, b**). Data are represented as mean ± SD.

Supplementary Fig 7 Flow cytometry gating strategy

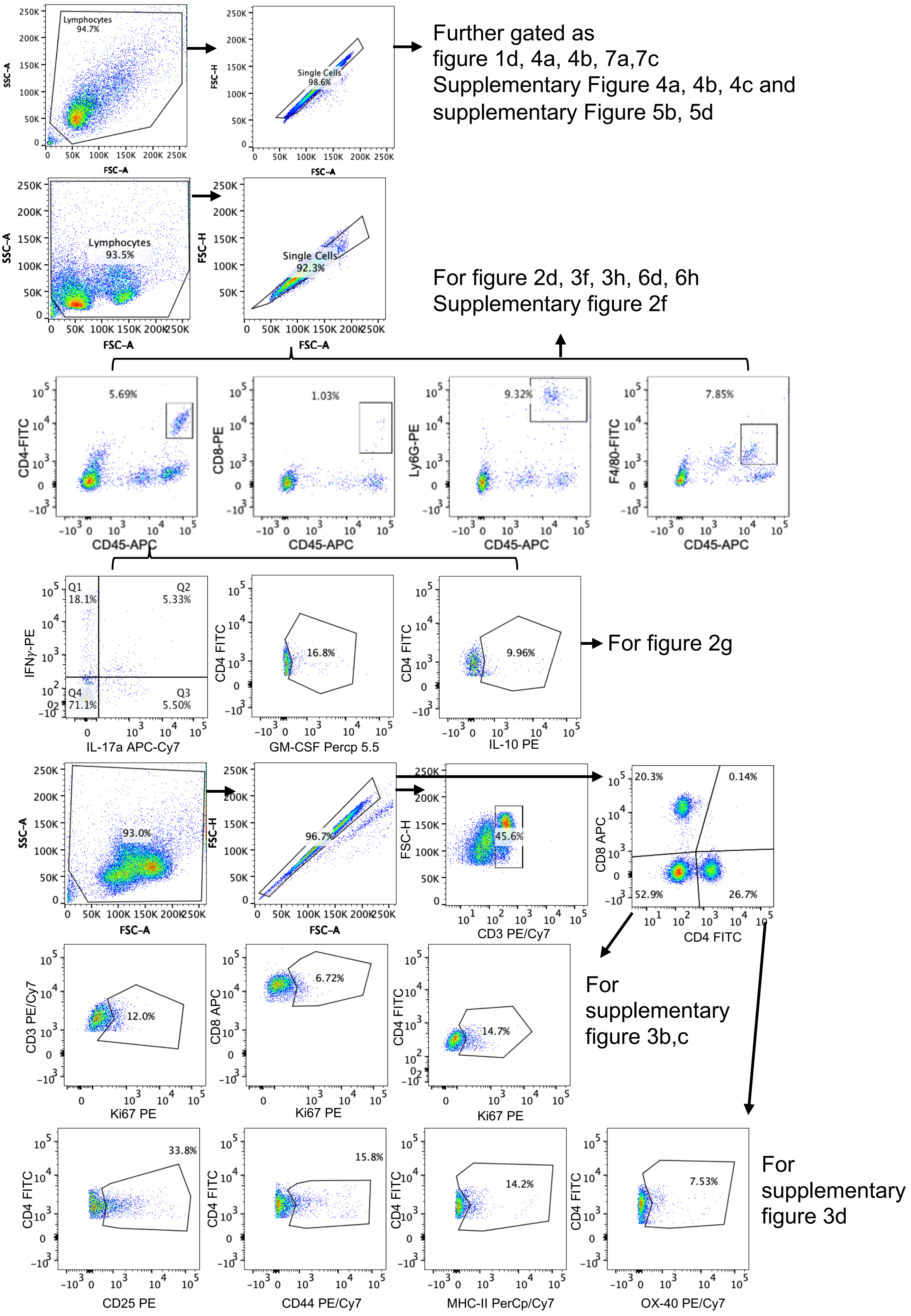

Supplementary Table 1 Gene-specific primers used in qRT-PCR experiments.

**Gene name**	**Forward primer 5'->3'**	**Reverse Primer 5'->3'**
Clec4e	catcccaccacacagagaga	ggtgatgaaacagccactga
Clec7a	catcgtctcaccgtattaatgcat	cccagaaccatggccctt
Clec4n	ttcttacttcctgggtctttcg	aacacaccgctcttctgga
Clec4d	gctggaagaatcccaaatga	aaagcaagcactaaggaacgag
Il17a	gtccagggagagcttcatctg	cttggcctcagtgtttggac
Ifng	ctcatggctgtttctggctg	ccttttgccagttcctccag
Tnf	ccaccacgctcttctgtcta	gatctgagtgtgagggtctgg
Csf2	ctaacatgtgtgcagacccg	gtctggtagtagctggctgtc
Ccl20	cacaagacagatggccgatg	cccttttcacccagttctgc
Cxcl1	ccagagcttgaaggtgttgc	tgaaccaagggagcttcagg
Cxcl9	aaacctgcctagatccggac	cgactttggggtgttttggg
Mmp9	cgacatagacggcatccagt	gataggccgtgggaggtatag
Il1b	ccatcctctgtgactcatggg	tcagctcatatgggtccgac
Il18	gccgacttcactgtacaacc	gtctggtctggggttcactg
Nlrp3	ggatatctctcccgcatctcc	gaggtccacactctcacctag
Il1r1	cccgaggtccagtggtataag	cttcagccacattcctcacc
