# Supplementary material for: TH17 cells promote CNS inflammation by sensing danger signals via Mincle
Source: Nat Commun. 2022 May 3;13:2406. doi: 10.1038/s41467-022-30174-1 (PMC9064974; doi:10.1038/s41467-022-30174-1)
Supplement: Supplementary file 3 — Reporting Summary [file 41467_2022_30174_MOESM3_ESM.pdf]

nature portfolio

Corresponding author(s): Laura E. Nagy

Last updated by author(s): Mar 11, 2022

Reporting Summary

Nature Portfolio wishes to improve the reproducibility of the work that we publish. This form provides structure for consistency and transparency in reporting. For further information on Nature Portfolio policies, see our Editorial Policies and the Editorial Policy Checklist.

Statistics

For all statistical analyses, confirm that the following items are present in the figure legend, table legend, main text, or Methods section.

n/a	Confirmed	
☐	☒	The exact sample size (*n*) for each experimental group/condition, given as a discrete number and unit of measurement
☐	☒	A statement on whether measurements were taken from distinct samples or whether the same sample was measured repeatedly
☐	☒	The statistical test(s) used AND whether they are one- or two-sided<br>*Only common tests should be described solely by name; describe more complex techniques in the Methods section.*
☒	☐	A description of all covariates tested
☐	☒	A description of any assumptions or corrections, such as tests of normality and adjustment for multiple comparisons
☐	☒	A full description of the statistical parameters including central tendency (e.g. means) or other basic estimates (e.g. regression coefficient) AND variation (e.g. standard deviation) or associated estimates of uncertainty (e.g. confidence intervals)
☐	☒	For null hypothesis testing, the test statistic (e.g. *F*, *t*, *r*) with confidence intervals, effect sizes, degrees of freedom and *P* value noted<br>*Give P values as exact values whenever suitable.*
☒	☐	For Bayesian analysis, information on the choice of priors and Markov chain Monte Carlo settings
☒	☐	For hierarchical and complex designs, identification of the appropriate level for tests and full reporting of outcomes
☒	☐	Estimates of effect sizes (e.g. Cohen's *d*, Pearson's *r*), indicating how they were calculated

Our web collection on statistics for biologists contains articles on many of the points above.

Software and code

Policy information about availability of computer code

Data collection	BD FacsDica V8.0.1 was used for Flow collection
Data analysis	All the image quantification analyses were performed using ImageJ software. GraphPad Prism 7 software was used for all graphical and statistical analysis. FlowJo™v10.4 was used for Flow data analysis.

For manuscripts utilizing custom algorithms or software that are central to the research but not yet described in published literature, software must be made available to editors and reviewers. We strongly encourage code deposition in a community repository (e.g. GitHub). See the Nature Portfolio guidelines for submitting code & software for further information.

Data

Policy information about availability of data

All manuscripts must include a data availability statement. This statement should provide the following information, where applicable:

- Accession codes, unique identifiers, or web links for publicly available datasets
- A description of any restrictions on data availability
- For clinical datasets or third party data, please ensure that the statement adheres to our policy

The source data underlying Fig. 1-7, Supplementary Fig.1-6 is provided as a Source Data file. Other data that support the study are available from the corresponding author(Lix@ccf.org).

Field-specific reporting

Please select the one below that is the best fit for your research. If you are not sure, read the appropriate sections before making your selection.

☒ Life sciences ☐ Behavioural & social sciences ☐ Ecological, evolutionary & environmental sciences

For a reference copy of the document with all sections, see nature.com/documents/nr-reporting-summary-flat.pdf

Life sciences study design

All studies must disclose on these points even when the disclosure is negative.

Sample size

The sample size was chosen to assure significant statistical differences and reproducibility of the results. Also, the maximum number of available mice for each experiment was used respecting the guidelines of animal welfare. At least, six mice were used in each experiment. For the experiments performed with cells, no sample size calculation was performed and three independent experiments were performed.

Data exclusions

No data was excluded.

Replication

Every experiment shown as repeated at least three times as indicated in the figure legends. All of replicate experiments produced consistent, statistically significant data, as indicated in figure legends.
For quantification of histological data, 3 views per slide, 3 sections per mouse (n=5) were included in the analysis.

Randomization

To prevent bias, analyzed samples did not bear any information about the mouse genotype, diet or treatment performed. Analyzes were done in a blinded manner without taking into account to which experimental groups the data belong to. Only age-matched mice were used for the study. Littermates of the same sex were randomly assigned to either experimental or control groups (described in Methods).

Blinding

For in vitro experiments, blinding was not applied, since all the samples were processed and analyzed with the same protocol and phenotype are distinct for different genotype. For in vivo experiments of EAE mice, investigator were blinded to group allocation during data collection and analysis.

Reporting for specific materials, systems and methods

We require information from authors about some types of materials, experimental systems and methods used in many studies. Here, indicate whether each material, system or method listed is relevant to your study. If you are not sure if a list item applies to your research, read the appropriate section before selecting a response.

Materials & experimental systems

n/a	Involved in the study	
☐	☒	Antibodies
☒	☐	Eukaryotic cell lines
☒	☐	Palaeontology and archaeology
☐	☒	Animals and other organisms
☒	☐	Human research participants
☒	☐	Clinical data
☒	☐	Dual use research of concern

Methods

n/a	Involved in the study	
☒	☐	ChIP-seq
☐	☒	Flow cytometry
☒	☐	MRI-based neuroimaging

Antibodies

Antibodies used

Anti-Mincle (1:1000, 1B6) was purchased from MBL. Anti-MCL(1:1000, PA5-102645) was purchased from Thermo Fisher. Anti-ASC (1:1000, N-15-R) was purchased from Santa Cruz Biotechnology. Anti-ASC (1:1000, 2EI-7) and anti-FcRγ(1:1000, 06-727) were purchased from Millipore. Anti-IL-1β (1:1000, AF-401-NA) was purchased from R&D. Anti-Caspase8 (1:1000, 4927, 1G12 and PA1-29159) were purchased from Cell Signaling Technology. Anti-NLRP3 (1:500, H-66) was purchased from SANT CRUZ Biotechnology. Anti-Actin (1:5000, A-2228) was purchased from Sigma. Anti-Ki67 (1:1000, ab15580) was purchased from Abcam. Anti-CD45-APC (1:500, 103112), Anti-F4/80-FITC (1:200, 123108) Anti-mouse-CD25-PE (1:300, PC61, 102008). anti-mouse/human CD44-PE/Cy7 (1:300, IM7, 103030), anti-mouse MCH-II-PerCP/Cy5.5 (1:300, M5/114.15.2, 107626), anti-mouse CD134/OX40-PE/Cy7, OX-86, 119415), anti-mouse GM-CSF-Percp5.5 (1:300, MP1-22E9, 505409), anti-mouse IL-10-PE (1:300, JES5-16E3, 505007) were purchased from Biolegend. Anti-CD4-FITC (1:200, L3T4), Anti-Ly6C-PE (1:300, HK1.4), Anti-IFN-γ-FITC (1:200, XMG1.2), Anti-CD3 (1:1000, 145-2C11), Anti-CD28 (1:1000, 37.51), Anti-IL-4-FITC (1:200, BVD6-24G2) and Anti-FOXP3-PE (1:300, FJK-16S) were purchased from eBioscience. Anti-IL-17A-PE (1:300, 559502), Anti-CD8-PE (1:300, 553041) and Anti-Ly6G-PE (1:300, 1A8) were purchased from BD.

Validation

1.Rat anti-mosue Mincle (1:1000, 1B6, D266-3) was purchased from MBL. https://www.mblbio.com/bio/g/dtl/A/?pcd=D266-3
2.Rabbit anti-mouse/human/rat MCL(1:1000, PA5-102645) was purchased from Thermo Fisher. https://www.thermofisher.com/antibody/product/CLEC4D-Antibody-Polyclonal/PA5-102645
3.Rabbit anti-mouse ASC (1:1000, N-15-R, sc-22514R) was purchased from Santa Cruz Biotechnology. https://www.scbt.com/p/asc-

antibody-n-15#thumbcarousel
4.Mouse anti-mouse/human ASC (1:1000, 2EI-7, 040-147) were purchased from Millipore.
https://www.emdmillipore.com/US/en/product/Anti-ASC-Antibody-clone-2EI-7,MM_NF-04-147
5.Rabbit anti-human/mouse/rat FcRγ(1:1000, 06-727) were purchased from Millipore.
https://www.emdmillipore.com/US/en/product/Anti-FcRI-Antibody-subunit,MM_NF-06-727?ReferrerURL=https%3A%2F%2Fwww.google.com%2F
6.Goat anti-mouse IL-1β (1:1000, AF-401-NA) was purchased from R&D.
https://www.rndsystems.com/products/mouse-il-1beta-il-1f2-antibody_af-401-na
7.Rabbit anti-mouse Caspase8 (1:1000, 9429) were purchased from Cell Signaling Technology.
https://www.cellsignal.com/products/primary-antibodies/cleaved-caspase-8-asp387-antibody-mouse-specific/9429
8.Rat anti-mouse Caspase8 (1:1000, 1G12, ALX-804-447-C100) were purchased from Enzo.
https://www.enzolifesciences.com/ALX-804-447/caspase-8-mouse-monoclonal-antibody-1g12/
9.Rabbit anti-mouse NLRP3 (1:500, H66, sc-66846) was purchased from SANT CRUZ Biotechnology.
https://www.scbt.com/p/cryopyrin-antibody-h-66
10.Mouse anti-mouse Actin (1:5000, AC74, A-2228) was purchased from Sigma. https://www.sigmaaldrich.com/US/en/product/sigma/a2228
11.Rabbit anti-mouse/human Ki67 (1:1000, ab15580) was purchased from Abcam.
https://www.abcam.com/ki67-antibody-ab15580.html
12.Rat anti-mouse CD45-APC (1:1000, 30F11, 103112) was purchased from Biolegend.
https://www.biolegend.com/en-us/products/apc-anti-mouse-cd45-antibody-97
13.Rat anti-mouse F4/80-FITC (1:200, BM8, 123108) was purchased from Biolegend.
https://www.biolegend.com/en-us/products/fitc-anti-mouse-f4-80-antibody-4067
14.Rat anti-mouse-CD25-PE (1:300, PC61, 102008) was purchased from Biolegend.
https://www.biolegend.com/en-us/products/pe-anti-mouse-cd25-antibody-424
15.Rat anti-mouse/human CD44-PE/Cy7 (1:300, IM7, 103030) was purchased from Biolegend.
https://www.biolegend.com/en-us/products/pe-cyanine7-anti-mouse-human-cd44-antibody-3932
16.Rat anti-mouse MCH-II-PerCP/Cy5.5 (1:300, M5/114.15.2, 107626) was purchased from Biolegend.
https://www.biolegend.com/en-us/products/percp-cyanine5-5-anti-mouse-i-a-i-e-antibody-4282
17.Rat anti-mouse CD134/OX40-PE/Cy7, OX-86, 119415) was purchased from Biolegend.
https://www.biolegend.com/en-us/products/pe-cyanine7-anti-mouse-cd134-ox-40-antibody-12101
18.Rat anti-mouse GM-CSF-Percp5.5 (1:300, MP1-22E9, 505409) was purchased from Biolegend.
https://www.biolegend.com/en-us/products/percp-cyanine5-5-anti-mouse-gm-csf-antibody-8754
19.Rat anti-mouse IL-10-PE (1:300, JES5-16E3, 505007) was purchased from Biolegend.
https://www.biolegend.com/en-us/products/pe-anti-mouse-il-10-antibody-944
20.Rat anti-CD4-FITC (1:200, L3T4, 11-0041-82) was purchased from eBioscience. https://www.thermofisher.com/antibody/product/CD4-Antibody-clone-GK1-5-Monoclonal/11-0041-82
21. Rat anti-Ly6C-PE (1:300, HK1.4, 12-5932-82) was purchased from eBioscience.
https://www.thermofisher.com/antibody/product/Ly-6C-Antibody-clone-HK1-4-Monoclonal/12-5932-82
22. Rat anti-IFN-γ-APC (1:200, XMG1.2, 11-7311-82) was purchased from eBioscience.
https://www.thermofisher.com/antibody/product/IFN-gamma-Antibody-clone-XMG1-2-Monoclonal/17-7311-82
23. Rat Anti-CD3 (1:1000, 145-2C11, 16-0031-82) was purchased from eBioscience.
https://www.thermofisher.com/antibody/product/CD3e-Antibody-clone-145-2C11-Monoclonal/16-0031-82
24. Rat anti-mouse CD28 (1:1000, 37.51, 16-0281-82) was purchased from eBioscience.
https://www.thermofisher.com/antibody/product/CD28-Antibody-clone-37-51-Monoclonal/16-0281-82
25. Rat anti-mouse IL-4-FITC (1:200, BVD6-24G2, 11-7042-82) was purchased from eBioscience.
https://www.thermofisher.com/antibody/product/IL-4-Antibody-clone-BVD6-24G2-Monoclonal/11-7042-82
26. Rat anti-mouse FOXP3-PE (1:300, FJK-16S, 12-5773-82) was purchased from eBioscience.
https://www.thermofisher.com/antibody/product/FOXP3-Antibody-clone-FJK-16s-Monoclonal/12-5773-82
27. Rat anti-mosue IL-17A-PE (1:300, TC11-18H10, 559502) was purchased from BD.
https://www.bdbiosciences.com/en-au/products/reagents/flow-cytometry-reagents/research-reagents/single-color-antibodies-ruo/pe-rat-anti-mouse-il-17a.559502
28. Rat anti-mouse CD8-PE (1:300, 53-5.8, 553041) was purchased from BD.
https://www.bdbiosciences.com/en-au/products/reagents/flow-cytometry-reagents/research-reagents/single-color-antibodies-ruo/pe-rat-anti-mouse-cd8b-2.553041
29. Rat anti-mouse Ly6G-PE (1:300, 1A8, 551461) was purchased from BD.
https://www.bdbiosciences.com/en-eu/products/reagents/flow-cytometry-reagents/research-reagents/single-color-antibodies-ruo/pe-rat-anti-mouse-ly-6g.551461
30. Rat anti-mouse Ki67PE (1:300, 16A8, 652404) was purchased from Biolegend.
https://www.biolegend.com/en-us/search-results/pe-anti-mouse-ki-67-antibody-8134
31. Rat anti-mouse CD3 PE/Cy7(1:300, 17A2, 100220) was purchased from Biolegend.
https://www.biolegend.com/en-gb/products/pe-cyanine7-anti-mouse-cd3-antibody-6060?GroupID=BLG242
32. Rat anti-mouse CD8 APC(1:300, 53-6.7,100712) was purchased from Biolegend.
https://www.biolegend.com/en-us/search-results/apc-anti-mouse-cd8a-antibody-150

Animals and other organisms

Policy information about studies involving animals; ARRIVE guidelines recommended for reporting animal research

Laboratory animals

Only adult female mice, aged 10-12 weeks were used for this study.(Cg)-Tg(Lck-Cd1d1)1Aben/J, B6 (Cg)-Tg(CD4-cre)1Cwi1/BfluJ, B6.129P2-Lyz2tm1(cre)Ifo/J and B6J.B6N(Cg)-Cx3cr1tm1.1(cre)Jung/J mice (C57BL/6 background), Rag1-/-, Nlrp3-/-, Il1b-/- and Caspase1/11 -/- were purchased from Jackson Laboratory (stock number 019418, 022071, 004781, 025524, 002216, 021302, 034447 and 016621) Asc flox/flox and Mincleflox/flox mice were generated by our lab.
These mice were housed in SPF condition, the ambient temperaqture is between 20-25°C, the humidity is between 40-70%, and the

	enviromental light/dark cycle is 12h light and 12h dark.<br>ASC Flox mice Martin, B.N. et al. T cell-intrinsic ASC critically promotes T(H)17-mediated experimental autoimmune encephalomyelitis. Nat Immunol 17, 583-592 (2016).
Wild animals	This study did not involve wild animals.
Field-collected samples	This study did not involve field-collected samples.
Ethics oversight	All procedures using animals were approved by the Cleveland Clinic Institutional Animal Care and Use Committee.

Note that full information on the approval of the study protocol must also be provided in the manuscript.

Flow Cytometry

Plots

Confirm that:

☒ The axis labels state the marker and fluorochrome used (e.g. CD4-FITC).

☒ The axis scales are clearly visible. Include numbers along axes only for bottom left plot of group (a 'group' is an analysis of identical markers).

☒ All plots are contour plots with outliers or pseudocolor plots.

☒ A numerical value for number of cells or percentage (with statistics) is provided.

Methodology

Sample preparation	Spleen or lymph nodes were isolated from mice, and wre smashed using 70uM strainer (red blood cells were lysised) to get the single cell solution for flow cytometry staining. For the isolation and analysis of CNS inflammatory cells, brains were homogenized in ice cold tissue grinders, filtered through a 100uM cell strainer and the cells collected by centrifugation at 350g for 5min at 4C. Cells were resuspended in 10ml of 30% Percoll and load on 70% Percoll cushion, followed by a 800g centrifuge for 30min. Cells at the 30-70% interface were collected and were subjected to flow cytometry.
Instrument	BD LSRFortessa
Software	BD FACSDiva™ v8.0.1 Software and FlowJo™v10.4 Software
Cell population abundance	For spleen and lymph nodes cell analysis, at least 30,000 CD4+ cells were analyzed. For the CNS infiltrated cells, all of the cells isolated from brain were analyzed.
Gating strategy	For all Flow cytometry analysis, population corresponding to viable cells were gated on FSC/SSC. Singlet were gated using FSC-A/FSC-H. Example gating strategy in Supplementray figure.

☒ Tick this box to confirm that a figure exemplifying the gating strategy is provided in the Supplementary Information.
